# Mitochondrial Dysfunction Leads to Deconjugation of Quercetin Glucuronides in Inflammatory Macrophages

**DOI:** 10.1371/journal.pone.0080843

**Published:** 2013-11-19

**Authors:** Akari Ishisaka, Kyuichi Kawabata, Satomi Miki, Yuko Shiba, Shoko Minekawa, Tomomi Nishikawa, Rie Mukai, Junji Terao, Yoshichika Kawai

**Affiliations:** 1 Faculty of Food Culture, Department of Nutrition, Kurashiki Sakuyo University, Kurashiki, Japan; 2 Department of Bioscience, Faculty of Biotechnology, Fukui Prefectural University, Fukui, Japan; 3 Department of Food Science, Graduate School of Nutrition and Biosciences, The University of Tokushima, Tokushima, Japan; 4 Laboratory of Food and Biodynamics, Graduate School of Bioagricultural Sciences, Nagoya University, Nagoya, Japan; Instituto de Química, Universidade de São Paulo, Brazil

## Abstract

Dietary flavonoids, such as quercetin, have long been recognized to protect blood vessels from atherogenic inflammation by yet unknown mechanisms. We have previously discovered the specific localization of quercetin-3-*O*-glucuronide (Q3GA), a phase II metabolite of quercetin, in macrophage cells in the human atherosclerotic lesions, but the biological significance is poorly understood. We have now demonstrated the molecular basis of the interaction between quercetin glucuronides and macrophages, leading to deconjugation of the glucuronides into the active aglycone. *In vitro* experiments showed that Q3GA was bound to the cell surface proteins of macrophages through anion binding and was readily deconjugated into the aglycone. It is of interest that the macrophage-mediated deconjugation of Q3GA was significantly enhanced upon inflammatory activation by lipopolysaccharide (LPS). Zymography and immunoblotting analysis revealed that β-glucuronidase is the major enzyme responsible for the deglucuronidation, whereas the secretion rate was not affected after LPS treatment. We found that extracellular acidification, which is required for the activity of β-glucuronidase, was significantly induced upon LPS treatment and was due to the increased lactate secretion associated with mitochondrial dysfunction. In addition, the β-glucuronidase secretion, which is triggered by intracellular calcium ions, was also induced by mitochondria dysfunction characterized using antimycin-A (a mitochondrial inhibitor) and siRNA-knockdown of Atg7 (an essential gene for autophagy). The deconjugated aglycone, quercetin, acts as an anti-inflammatory agent in the stimulated macrophages by inhibiting the c-Jun N-terminal kinase activation, whereas Q3GA acts only in the presence of extracellular β-glucuronidase activity. Finally, we demonstrated the deconjugation of quercetin glucuronides including the sulfoglucuronides *in vivo* in the spleen of mice challenged with LPS. These results showed that mitochondrial dysfunction plays a crucial role in the deconjugation of quercetin glucuronides in macrophages. Collectively, this study contributes to clarifying the mechanism responsible for the anti-inflammatory activity of dietary flavonoids within the inflammation sites.

## Introduction

Polyphenols are a large family of natural compounds widely distributed in plant foods, and have been linked to improved human health through reduced chronic diseases, especially cardiovascular diseases. Among them, flavonoids are the most abundant polyphenols in our diets. Flavonoids are characterized by a phenylbenzopyran chemical structure, which includes a C_15_ (C_6_-C_3_-C_6_) skeleton joined to a chroman ring (benzopyran moiety). In 1936, Rusznyak and Szent-Gyorygi determined that citrus flavonoids reduced capillary fragility and permeability in blood vessels [1,2]. Thereafter, a large number of biological activities of flavonoids (also called “vitamin P”) were described that are generally believed to be beneficial for good health. Quercetin (3,5,7,3’,4’-pentahydroxyflavone) is the major flavonol-type flavonoid and is particularly abundant in onion [3] and tea [4], which represent the major sources of flavonoids in the Dutch diet [5]. Epidemiological evidence links diets rich in quercetin and other flavonoids with the decreased incidence of cardiovascular, neoplastic, and neurodegenerative diseases [6-13].

In contrast, some papers strongly suggested the pro-oxidative and pro-inflammatory properties of excess polyphenols *in vitro* [14-17], whereas such a toxicity has not yet been revealed *in vivo*. The putative role of dietary flavonoids in the protection of chronic diseases [18] without toxicity or side effects suggests the presence of a distinct pathway between the detoxification and biological activity *in vivo*. After oral intake, absorbed flavonoids are metabolized to glucuronides and/or sulfates by phase II reactions, and some (if containing the catecholic moiety) are further methylated by catechol-*O*-methyltransferase (COMT), and therefore non-conjugated aglycones are scarcely detected in the human plasma [19]. The flavonoid metabolites have been detected in tissues and organs [20-22], while only limited information is presently available on their localization and molecular dynamics underlying the biological activity of flavonoids *in vivo*. Kamada et al. have previously demonstrated the accumulation of quercetin metabolites in the aorta of quercetin-fed hypercholesterolemic rabbits, where the peroxidation of aortic cholesterols was significantly inhibited [23]. More recently, we have immunohistochemically shown that quercetin-3-*O*-glucuronide (Q3GA), a major phase II metabolite of quercetin in rats and humans [19,24], specifically accumulates in the macrophage-derived foam cells in the human atherosclerotic arteries [25]. Similar localization of a tea catechin, epicatechin gallate, in foamy macrophages has also been demonstrated in the human aorta [26]. These results suggest that the macrophages could be a target of flavonoids and their metabolites *in vivo*. However, the molecular mechanisms for the accumulation of flavonoids and their metabolites in macrophages and the biological consequences still remain unclear. In this study, we provided a novel pathway for the selective biological activity of quercetin metabolites in macrophages within the inflamed sites and tissues. We demonstrated the involvement of mitochondrial dysfunction in macrophages in the anti-inflammatory activity of quercetin glucuronides. 

## Materials and Methods

### Materials

Quercetin dehydrate, sulfatase H-1, 4,4’-diisothiocyanatostilbene-2,2’-disulfonic acid (DIDS), and 4-acetamido-4’-isothiocyanatostilbene-2,2’-disulfonic acid (SITS) were purchased from the Sigma-Aldrich Co. (St. Louis, MO, USA). Isorhamnetin (3’-*O*-methylquercetin), quercetin-3-*O*-glucoside, and quercetin-3-*O*-galactoside were obtained from Extrasynthese (Genay, France). Q3GA was chemically synthesized as previously reported [24]. The rabbit polyclonal antibody to β-glucuronidase was obtained from ProteinTech Group, Inc. (Chicago, USA). The goat polyclonal antibody to cyclooxygenase-2 (COX-2) was obtained from Santa Cruz Biotechnology, Inc. (Santa Cruz, CA, USA). Rabbit polyclonal antibodies to GAPDH, phospho-JNK, JNK, phospho-p38, p38, phospho-ERK, ERK, IκB, NFκB, phospho-NFκB, phospho-c-Jun, and c-Jun were obtained from Cell Signaling Technology (Beverly, MA, USA). *O,O*’-Bis(2-aminophenyl)ethyleneglycol-*N,N,N*’,N’-tetraacetic acid, tetraacetoxymethyl ester (BAPTA-AM) was obtained from Invitrogen (Carlsbad, CA, USA). Fluo4-AM was obtained from Dojindo Laboratories (Kumamoto, Japan).

### Cell culture

RAW264 was obtained from the Riken Cell Bank (Ibaraki, Japan). J774-1 and THP-1 were obtained from the Cell Resource Center for Biomedical Research Institute of Development, Aging, and Cancer, Tohoku University (Sendai, Miyagi, Japan). RAW264 and J774-1 were cultured in Dulbecco’s modified Eagle’s medium (DMEM, Sigma) with 10% fetal bovine serum (FBS). THP-1 cells were cultured in RPMI1640 medium (Sigma) containing 10% FBS and differentiated into macrophage-like cells (*d*-THP-1) by exposure to 100 nM phorbol 12-myristate 13-acetate (PMA) for 3 days. Bovine aortic endothelial cells (BAECs, a kind gift from Dr. Yutaka Taketani, The University of Tokushima, Japan) [27] were cultured in Medium 199 (Sigma) containing 20% FBS. Human umbilical vein endothelial cells (HUVEC) were obtained from TOYOBO (Osaka, Japan) and cultured in Endothelial Cell Basal Medium (Cell Applications, Inc., CA, USA). The peritoneal macrophages were obtained from the peritoneal fluids of ICR mice (female, 6-9 weeks old; Japan SLC, Shizuoka, Japan) primed for 4 days by intraperitoneal injection of 2 ml of 10% proteose peptone. Five hours after seeding, the nonadherent cells were removed by washing, and the adherent monolayer was maintained in DMEM with 10% FBS. The RAW264/ρ0 cells were developed by the long-term culturing of the RAW264 cells in the medium containing ethidium bromide (50 ng/ml) and uridine (50 μg/ml). All media contain penicillin (100 μg/ml) and streptomycin (100 units/ml). 

### Analysis for cellular accumulation of quercetin derivatives

Cells (in 60-mm dish), grown to ~80% confluence, were treated with the quercetin derivatives in FBS-free medium. For the experiments using inhibitors of COMT or β-glucuronidase, the cells were treated with Q3GA (20 μM) in the presence of 3,5-dinitrocatechol (10 μM) or d-saccharic acid 1,4-lactone (1 mM). To obtain the continuous activity of d-saccharic acid 1,4-lactone due to its instability, it was added to the medium every 3 h. For the inhibition experiments for Q3GA accumulation, metabolic inhibitors, anion transporter inhibitors, or organic anion transporter substrates were added to the medium for 15 min followed by the incubation with Q3GA (50 µM) for 15 min.

After incubation, the cells were washed three times with Hank’s balanced salt solution (HBSS), scraped from the dish, and pelleted by centrifugation. An aliquot of the cell pellet was used for measuring the cell numbers or protein content. The quercetin derivatives were extracted from the cells into methanol/acetic acid (100:1) with sonication for 1 min. After centrifugation, the supernatants were collected, evaporated, and dissolved in HPLC solvent. Fifty µl of the sample was injected into an HPLC-electrochemical detection (ECD) system as described below. Statistical analysis was performed by two-tailed Student’s *t*-tests to determine the P values. The results are expressed as mean ±SD.

### High-performance liquid chromatography-electrochemical detection

Samples were injected into an HPLC-ECD system (ESA, Cambridge, MA) equipped with a TSK-gel ODS-80Ts column (4.6 × 150 mm, Tosoh Bioscience, Tokyo, Japan). The separation of the compounds was carried out by a gradient elution. Solvent A was 0.5% phosphoric acid, and solvent B was 100% acetonitrile containing 0.5% phosphoric acid. The gradient program was as follows: 0-10 min, 21% B; 10-20 min, linear gradient to 44% B; 20-25 min, linear gradient to 21% B; 25-30 min, hold; flow rate, 0.8 ml/min. Electrochemical detection was performed using a coulometric electrode at 150 mV. Quantitation of the quercetin compounds was performed using standard curves developed by the peak areas of authentic compounds (Q3GA, quercetin, and isorhamnetin).

### Preparation of monoclonal antibody to DIDS

The anti-DIDS monoclonal antibody (mAb16D4) was raised by immunizing BALB/c mice (female, 6 weeks old; Japan SLC, Shizuoka, Japan) with DIDS-modified keyhole limpet hemocyanin (KLH). The immunogen was prepared by incubating KLH (2 mg/ml) with DIDS (20 mM) at 37 °C for 2.5 h and then dialyzed against PBS over 24 h at 4 °C. After repeated immunization, the antibody titer against the DIDS-modified bovine serum albumin (BSA) was evaluated by enzyme-linked immunosorbent assay (ELISA) according to the previously reported protocol [25]. Development of hybridomas, screening, and cloning were performed as previously described [25]. We finally obtained a monoclonal hybridoma (16D4) and prepared the immunoglobulins from the culture supernatant using the ammonium sulfate precipitation method.

### Trypsinization of cell surface proteins

The cells were treated with DIDS or quercetin derivatives in the FBS-free DMEM medium for 15 min and washed three times with HBSS. The cells were then treated with a trypsin solution (TrypLE Express, Invitrogen) at room temperature for 1 min. The trypsinization was terminated by adding excess DMEM medium. The cells were washed three times with HBSS and the accumulated quercetin derivatives were extracted and analyzed as already described.

### Zymography for β-glucuronidase activity in the medium

The cultured medium was removed from the dishes. After centrifugation, the supernatants were collected and then mixed with a non-reducing sample buffer (0.25 M Tris-HCl, pH 6.8, 25% glycerol, 0.025% bromophenol blue). The samples were separated on 4-20% Tris-Glycine gel (Invitrogen) followed by incubating gel in 2.5% Triton X-100 for 1 h to remove the SDS. The gel was washed twice with purified water and incubated overnight in the substrate solution at 37 °C. The substrate solution was composed of 1 mM 5-bromo-4-chloro-3-indolyl-β-d-glucuronide (X-glucuronide, Sigma) in 0.1 M sodium acetate buffer (pH 5.0).

### β-Glucuronidase Activity

The enzyme activity in the medium was evaluated using Q3GA, X-glucuronide, or 4-methylumbelliferone glucuronide (MUG) as the substrate. For analysis using Q3GA as the substrate, the culture medium was removed from the dishes, centrifuged to remove the cells, and then incubated with 50 μM Q3GA at 37 °C for 1 h. After incubation, the formed aglycone was extracted twice with ethyl acetate, evaporated, and dissolved in the mobile phase for the HPLC-ECD analysis. Ten μl of the samples was injected into the HPLC-ECD system as already described. 

For analysis using X-glucuronide as the substrate, 37.5 μl of the medium was mixed with 75 μl of 0.1 M sodium acetate buffer (pH 5.0) and 12.5 μl of 10 mM X-glucuronide in a 96-well plate and incubated at 37 °C for 3.5 h. After incubation, the absorbance was measured at 590 nm using a microplate reader. The relative activity was expressed as the absorbance at 590 nm or as % increase vs control. For analysis using MUG as the substrate, 66 μl of the medium was mixed with 132 μl of 0.1 M sodium acetate buffer (pH 5) and 2 μl of 10 mM MUG in a 96-well plate and incubated at 37 °C for 1 h. After incubation, the fluorescence intensity was measured at 365 nm excitation and 450 nm emission using a fluorescence microplate reader (Varioskan Flash, Thermo). The relative activity was expressed as % increase in the fluorescence intensity vs control. Using the same protocol, the sulfatase activity in the medium was measured using 4-methylumbelliferone sulfate instead of MUG.

### Immunoblot analysis

The cells in 6 well dish were washed twice with HBSS and lysed with a radio-immunoprecipitation assay (RIPA) lysis buffer (50 mM Tris-HCl, pH 7.5, 150 mM NaCl, 1% Nonidet P-40, 0.25% sodium deoxycholate, 1 mM EDTA) containing protease/phosphatase inhibitors (Pierce, Rockford, IL, USA). The fractionation of the nuclear/cytoplasmic fractions was carried out according to the NE-PER Nuclear and Cytoplasmic Extraction Reagents (Pierce). The protein concentrations were determined by the BCA Protein Assay Kit (Pierce). The protein samples were mixed with a reducing sample buffer and boiled for 5 min. The samples (50 μg protein) were run on 10% SDS-polyacrylamide gels, transferred to a poly(vinylidene) fluoride membrane (Hybond-P, GE Healthcare) by a semi-dry transfer method. The blots were incubated with a blocking reagent (EzBlock, ATTO Corp., Tokyo, Japan) in TTBS (Tris-buffered saline containing 0.05% Tween 20) at room temperature for 30 min, washed three times with TTBS, and then treated with a primary antibody in 5% BSA in TTBS at 4 °C overnight. After washing, the blots were further incubated with the respective secondary antibodies conjugated with horseradish peroxidase in TTBS at room temperature for 1 h. After washing, the blots were treated with a SuperSignal West Pico enhanced chemiluminescence substrate (Thermo Scientific, Rockford, IL, USA) at room temperature for 5 min and visualized by a LAS-1000 Chemiluminescence Imager (Fuji Photo Film Company, Kanagawa, Japan).

### Lactate secretion

The medium was mixed with an equal volume of acetonitrile containing 0.1% formic acid and kept on ice for 10 min. After centrifugation at 12,000 g for 10 min, 25 μl of the supernatants or authentic lactate solution was mixed with 475 μl of aqueous 0.1% formic acid containing 400 μM [3-^13^C_1_]lactate (Santa Cruz Biotechnology) as an internal standard. Ten μl of the samples was analyzed by a liquid chromatography-tandem mass spectrometry (LC-MS/MS) system (API2000^TM^ LC/MS/MS system, AB Sciex) equipped with a TurboIonSpray^TM^ (Electrospray Ionization; ESI), an Agilent series 1100 HPLC pump and autosampler (Agilent Technologies, Inc.). The chromatographic separation was performed on a Scherzo SM-C18 column (2.0 x 75 mm, Imtakt Corporation, Kyoto, Japan) at a flow rate of 200 μl/min. Eluent A was 0.1% formic acid, whereas eluent B was methanol containing 0.1% formic acid. The gradient conditions were as follows: 0-2 min, 2%B; 2-8 min, a linear gradient from 2%B to 80%B. The mass spectrometric parameters in a negative ionization mode were optimized as follows using the built-in syringe pump: Declustering potential (DP) at -56V, Focusing potential (FP) at -250V, Entrance potential (EP) at -10V, Collision cell entrance potential (CEP) at -8V, Collision energy (CE) at -14 V, and Collision cell exit potential at -6V. Using flow injection analysis, ion source parameters were optimized as follows: Curtain gas at 50 psi, CAD 3, IonSpray voltage at -4500 V, Heater gas temperature at 400 °C, Nebulizer gas (GAS1) at 60 psi, and Turbo gas (GAS2) at 80 psi. The following multiple reaction monitoring transitions were monitored to determine the lactate: *m*/*z* 89 > 43, unlabeled lactate; and *m*/*z* 90 > 44, [3-^13^C_1_]lactate. The data were acquired and processed with Analyst 1.1^TM^ software (AB Sciex).

### Intracellular Ca^2+^ measurement

The intracellular calcium ion was measured using a Fluo4-AM probe. Briefly, cells in a 24-well plate were pre-treated with Fluo4-AM (5 μM) for 1 h, washed twice, and then treated with chemicals. After treatment, the fluorescence intensity was measured by a Typhoon scanner (GE healthcare).

### Transfection

The pcDNA3-Flag-JNK and the dominant-negative pcDNA3-Flag-JNK1a1 were obtained from Addgene (Cambridge, MA). These plasmids were transfected using Lipofectamine 2000 (Invitrogen) according to the suppliers’ standard protocols. Pre-designed and validated Stealth siRNAs were purchased from Invitrogen. Transfection of the plasmids and siRNAs was performed using Lipofectamine RNAiMAX (Invitrogen) according to the suppliers’ standard protocols.

### Animals

ICR mice were obtained from Japan SLC (Shizuoka, Japan). The mice were housed in a controlled room (temperature, 23 ± 1 °C; humidity, 45-50%; light-dark cycle, 12 h each; lights on, 8:00 AM). This study was performed according to the guidelines for the care and use of laboratory animals of The University of Tokushima Graduate School, Institute of Health Biosciences. The protocol was approved by the Committee on Animal Experiments of the University of Tokushima (permit number: 11030). All surgery was undertaken under anesthesia using sodium pentobarbital or ether. All efforts were made to minimize animal suffering and to reduce the number of animals used.

### Lipopolysaccharide-induced inflammation in mice

The ICR mice (male, 6-8 weeks old) were given free access to a purified diet as previously described [28]. For the *in vivo* inflammation experiments with quercetin administration, the mice were given free access to a purified diet (control group) or a purified diet containing 0.5% (w/w) quercetin (quercetin group) for 24 h followed by intraperitoneal injection of lipopolysaccharide (LPS, 3 mg/kg body weight in PBS) or PBS alone. The mice were sacrificed after 24 h of LPS injection with continuous free access to the quercetin diet. For analysis of the total quercetin metabolites in the tissues, 500 μl of the tissue homogenates (in PBS) were mixed with 250 μl sulfatase H-1 (4 mg/ml in a sodium acetate buffer, pH 5.0), which has both sulfatase and β-glucuronidase activity, and incubated at 37 °C for 90 min in the presence of 100 mM ascorbic acid. After incubation, the non-conjugated quercetins were extracted twice with ethyl acetate. For analysis of the non-conjugated quercetins, the tissue homogenates were directly extracted with ethyl acetate. To prevent the artificial deconjugation during sample preparation, d-saccharic acid 1,4-lactone (5 mM) was added to the homogenates. For analysis of the quercetin sulfates in the tissues, the tissue homogenates were treated with sulfatase H-1 in the presence of 5 mM d-saccharic acid 1,4-lactone. The total metabolites were estimated as the amounts of the aglycones in the homogenates treated with sulfatase H-1 minus the aglycones in the intact homogenates. The sulfate levels were estimated as increased amounts of the non-conjugated quercetins in the homogenates treated with sulfatase H-1 in the presence of the β-glucuronidase inhibitor. The plasma quercetin derivatives were also estimated by the same procedure for the tissues.

### Real-time RT-PCR

A quantitative real-time RT-PCR was performed using the TaqMan® gene expression assay and TaqMan® universal PCR master mix reagents (Applied Biosystems). The RT reaction was performed with 1 μg of total RNA and random primer using the high capacity cDNA reverse transcription kit (Applied Biosystems). The amplification of the PCR products was monitored by an Applied Biosystems 7500 real time PCR system. The reaction conditions for the RT and PCR were based on the protocols provided by Applied Biosystems. The relative levels of the gene expression for each sample were calculated using the comparative Ct method. The target gene (COX-2) expression in each sample was normalized to the GAPDH Ct values. Data are expressed as the means ± S.D. of three separate experiments.

## Results

### Interaction of Q3GA to macrophages

**Figure 1:**
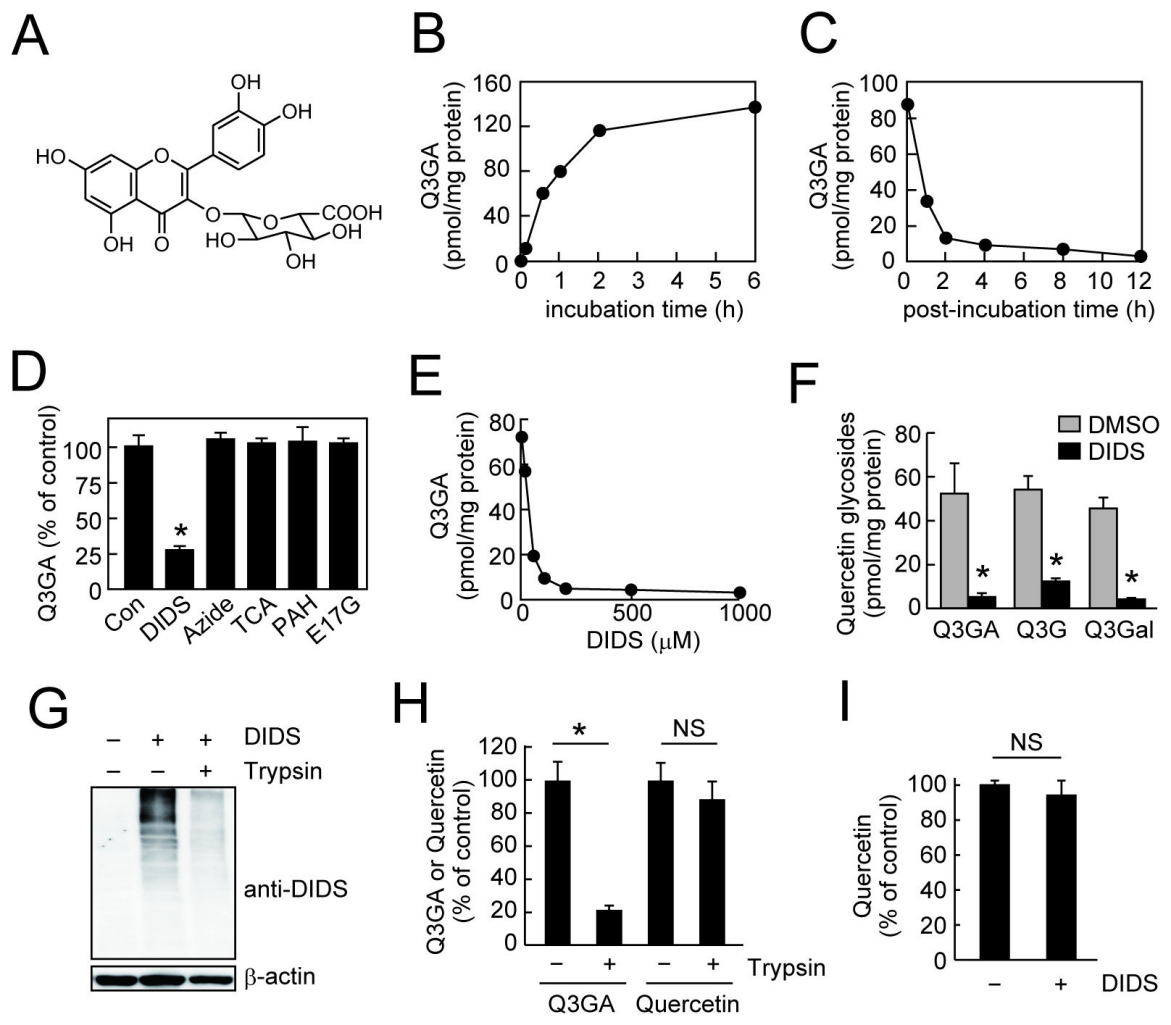
Cell-surface binding of Q3GA. (A) Chemical structure of quercetin-3-*O*-glucuronide (Q3GA). (B) Time-dependent accumulation of Q3GA in the RAW264 cells. Cells were treated with Q3GA (50 μM) for the indicated time periods. (C) time-dependent dissociation of Q3GA bound to RAW264 cells. Cells were treated with Q3GA (50 μM) for 1 h, washed, and then incubated in the fresh medium for indicated time periods. Data points represent duplicate determinations. (D) Inhibition of Q3GA accumulation in RAW264 cells by various transporter inhibitors. Cells were pretreated with inhibitors for 15 min, followed by Q3GA (50 μM) treatment for 15 min. The concentrations of inhibitors were as follows: DIDS, taurocholic acid (TCA), p-aminohippuric acid (PAH), and estradiol-17-β-glucuronide (E17G), 500 μM; sodium azide, 5 mM. (E) Dose-dependent inhibition of Q3GA accumulation in RAW264 cells by DIDS. Data points represent duplicate determinations. (F) Inhibition of the accumulation of quercetin-3-*O*-glycosides in RAW264 cells by DIDS. The DIDS-treated cells were incubated with each quercetin-3-*O*-glycoside (20 μM) for 15 min. Q3G, quercetin-3-*O*-glucoside; Q3Gal, quercetin-3-*O*-galactoside. (G) Anti-DIDS immunoreactivity of the lysates of the DIDS-treated RAW264 cells. Cells were treated with DIDS followed by a quick trypsinization for 1 min. β-Actin was detected as a positive control for intracellular proteins. (H) Effects of quick trypsinization of RAW264 cells on the accumulation of Q3GA and quercetin. Q3GA- or quercetin-treated cells were incubated with trypsin for 1 min. (I) Effect of DIDS treatment on the accumulation of quercetin. Data in all bar graphs are presented as the average ± S.D. (n=3). Asterisks indicate a significant difference (*p* < 0.05). NS, not statistically significant.

To investigate the biological consequences of the Q3GA accumulation in the macrophages, we first examined the cellular accumulation of Q3GA in the macrophages *in vitro*. The significant accumulation of Q3GA (see the chemical structure in Figure 1A) was indeed observed in the Q3GA-treated RAW264 cells (Figure 1B), while the accumulation was found to be reversible (Figure 1C), showing the non-covalent binding of Q3GA to the cellular components. We found that the accumulation of Q3GA in the cells was inhibited by the pre-treatment with stilbene disulfonate derivatives, DIDS (Figures 1 D and E) and SITS (data not shown), which are well-characterized anion transport inhibitors. The chemical properties of DIDS and SITS represent non-covalent anion binding *via* sulfonate groups followed by covalent binding with amino groups *via* isothiocyanate groups to the target proteins [29]. In contrast, several anion transporter substrates and a metabolic inhibitor, sodium azide, which inhibits ATP production, did not affect the accumulation of Q3GA (Figure 1D). These results suggest that Q3GA accumulates in the cells through anion binding to DIDS-sensitive proteins, but not through energy-dependent transporter systems. Not only the binding of Q3GA, the binding of both quercetin-3-*O*-glucoside and quercetin-3-*O*-galactoside, which are glycosides with a neutral sugar moiety, was also inhibited by DIDS (Figure 1F), strongly suggesting that the phenolic group(s) of quercetin moiety and not the glucuronic acid moiety, might be involved in the binding of Q3GA to the cellular proteins. The binding of Q3GA to the cells was inhibited by 40% at 4 °C (data not shown), also indicating the involvement of protein binding. 

To detect the DIDS-binding proteins, we newly developed a monoclonal antibody (mAb16D4) by immunizing mice with the DIDS-modified protein (Figure S1A). Immunoblot analysis demonstrated that the immunoreactivity of the DIDS-modified BSA was observed not only as a monomer, but also as higher molecular weight forms, showing the intermolecular crosslink of the protein *via* two isothiocyanate groups in the DIDS (Figure S1B). The DIDS-modified proteins were also detected in the lysate of the DIDS-treated RAW264 cells (Figure S1C). A quick trypsinization of the DIDS-treated RAW264 cells almost completely attenuated the DIDS-immunoreactivity (Figure 1G). In this experimental condition, the cell surface proteins, but not the intracellular proteins (such as β-actin), could be digested. These results suggested the binding of DIDS to the cell surface proteins. Similarly, the cell surface binding of Q3GA was demonstrated by the observation that its cellular accumulation was also inhibited by a quick trypsinization of the Q3GA-treated RAW264 cells (Figure 1H). In contrast, cellular accumulation of the quercetin aglycone, which is incorporated into cells presumably *via* simple diffusion, was inhibited neither by trypsinization (Figure 1H) nor by pretreatment with the DIDS (Figure 1I). The DIDS-sensitive cell surface binding of Q3GA was also observed in mouse peritoneal primary macrophages (data not shown).

**Figure 2:**
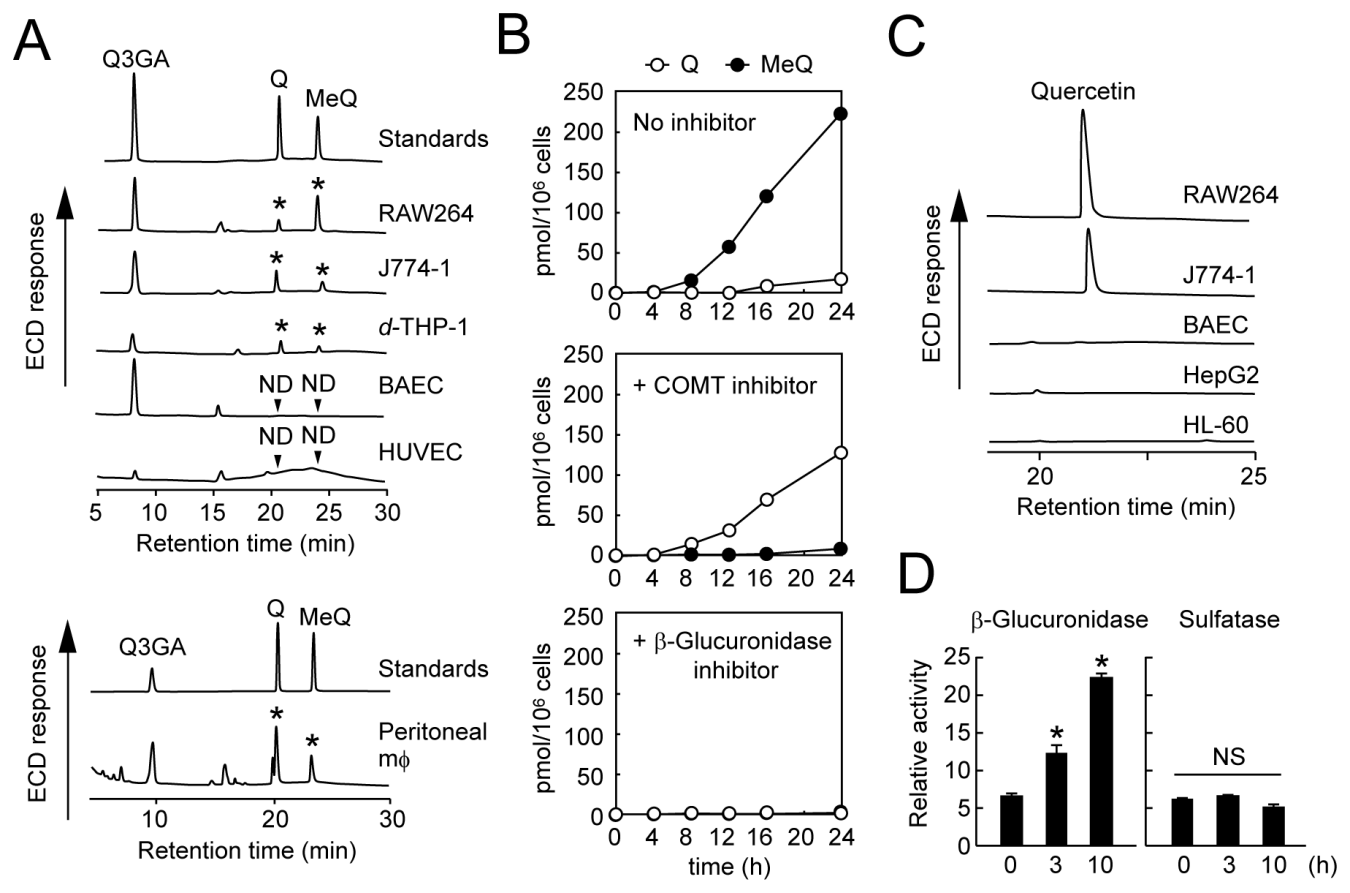
Accumulation and deconjugation of Q3GA in macrophage cells. (A) HPLC-ECD analysis of quercetin derivatives in various cell lines (upper) and in mouse peritoneal macrophages (lower) treated with Q3GA (20 μM) for 8 h. Q, quercetin; MeQ, 3’- or 4’-*O*-methylquercetins. Asterisks indicate the peaks corresponding to Q or MeQ. ND, not detected. (B) Effects of inhibitors on accumulation of quercetin and methylquercetins during the treatment of RAW264 cells with Q3GA. *Top*, no inhibitor; *middle*, COMT inhibitor 3,5-dinitrocatechol; *bottom*, β-glucuronidase inhibitor d-saccharic acid 1,4-lactone. Data points represent duplicate determinations. (C) HPLC-ECD analysis of quercetin formed by the deconjugation of Q3GA in the cell-free conditioned medium. The conditioned medium was prepared after culturing each cell line for 24 h, and incubated with Q3GA (50 μM) at 37 °C for 1 h. (D) Relative enzyme activity of β-glucuronidase (left) and sulfatase (right) in the medium during culturing RAW264 cells, determined using 4-methylumbelliferyl substrates. Data in all bar graphs are presented as the average ± S.D. (n=3). Asterisks indicate a significant difference (*p* < 0.05). NS, not statistically significant.

As shown in Figure 2A and our previous report [25], not only Q3GA, but quercetin and methylquercetin(s) (i.e., 3’-methylquercetin and/or 4’-methylquercetin) were detected in the Q3GA-treated RAW264 cells. These non-conjugated derivatives were also detected in other macrophage-like cell lines (J774-1 and differentiated THP-1) and in the peritoneal primary macrophages after treatment with Q3GA, but not detected in the vascular endothelial cells (HUVEC and BAEC) and other cell lines from different tissues, as far as we tried (Table 1). The generation of quercetin and methylquercetins from Q3GA reflects the possible activity of β-glucuronidase and COMT in the macrophages. As shown in Figure 2B, the addition of a COMT inhibitor during the treatment of RAW264 cells with Q3GA resulted in the increased accumulation of quercetin instead of the methylquercetins and, in contrast, the addition of a β-glucuronidase inhibitor almost completely inhibited the accumulation of both quercetin and the methylquercetins. The methylquercetin-3-glucuronide could not be detected in the cell lysates (data not shown). Given that COMT is generally localized inside the cells [30] and that a hydrophilic β-glucuronidase inhibitor may act extracellularly, these results suggest that the β-glucuronidase-catalyzed deconjugation of Q3GA is the first step that occurs in the extracellular fluid or cell surface, followed by entering cells of the quercetin aglycone through simple diffusion, and by the methylation of intracellular quercetin catalyzed by COMT. Indeed, the β-glucuronidase activity was observed in the cell-free cultured medium (Figure 2C), indicating the secretion of β-glucuronidase activity from the macrophage cells. The enzymatic activity of sulfatase, another important deconjugation enzyme, was scarcely observed in the cultured medium of the RAW264 cells (Figure 2D). 

**Table 1:** The ability of various cell lines to deconjugate Q3GA into the aglycone.

Cells	Types	β-Glucuronidase activity
RAW264	mouse mϕ	+
J774-1	mouse mϕ	+
*d*-THP-1	human mϕ	+
peritoneal mϕ	mouse primary mϕ	+
BAEC	bovine aortic endothelium	‒
HUVEC	human umbilical vein endothelium	‒
HL-60	human leukemia	‒
HepG2	human hepatocarcinoma	‒
NIH-3T3	mouse fibrobrast	‒
Neuro-2A	mouse neuroblastoma	‒

Cells were treated with Q3GA (50 μM) in the fresh medium for 8 h. The accumulated quercetin derivatives were extracted from the cell lysates and analyzed by HPLC-ECD. When the aglycone (quercetin and/or methylquercetins) was detected, the β-glucuronidase activity of the cell line was expressed as “+” (if not, shown as “‒” ). Abbreviations: *d*-THP-1, PMA-differentiated THP-1; mϕ, macrophage.

### Deconjugation of quercetin glucuronides through mitochondrial dysfunction

**Figure 3:**
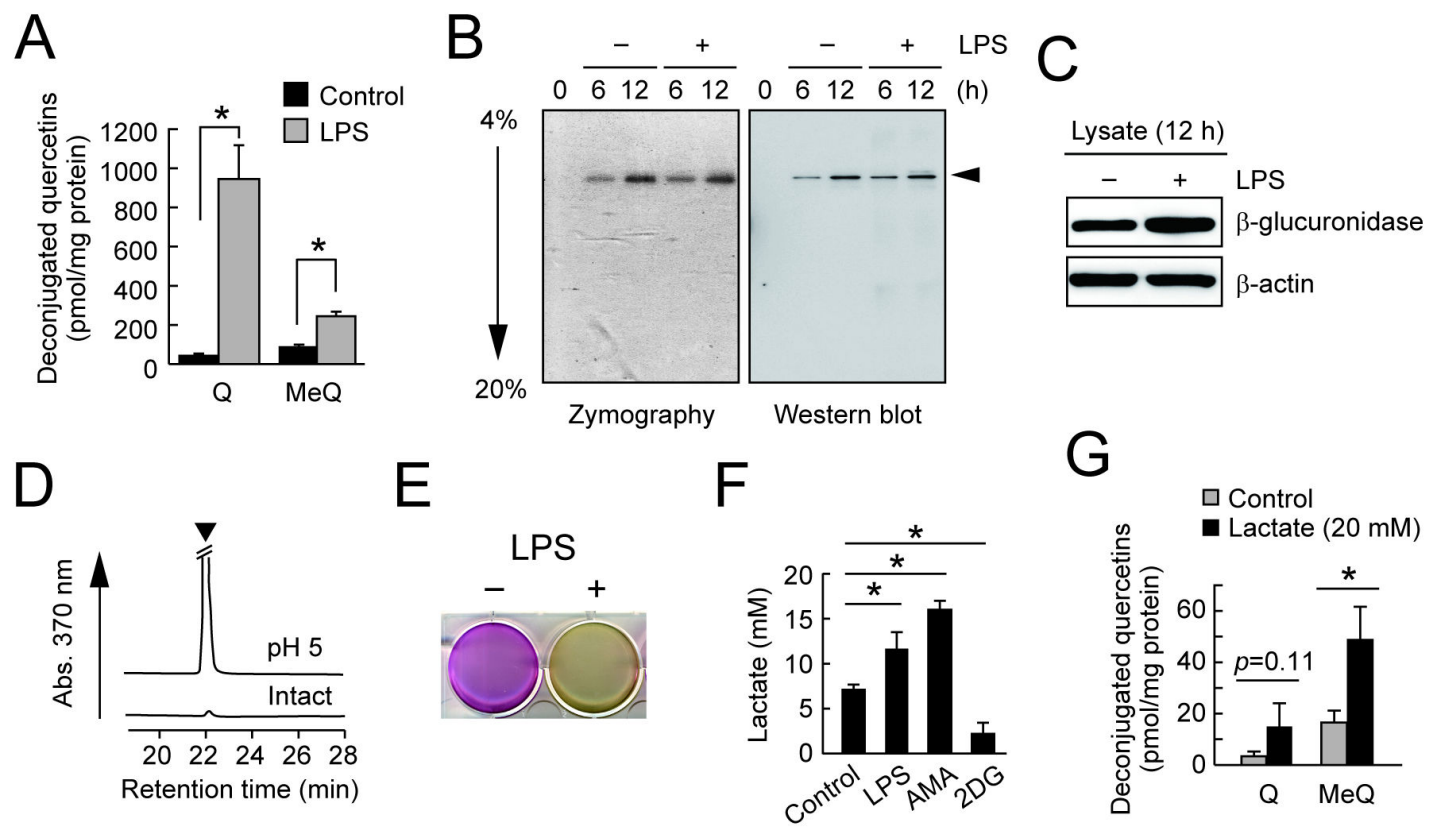
β-Glucuronidase activity in LPS-treated RAW264 cells. (A) Enhanced deconjugation of Q3GA in the LPS-stimulated RAW264 cells. Cells were pretreated with LPS (1 μg/ml) for 8 h followed by treatment with Q3GA (20 μM) for 1 h. The deconjugated quercetin derivatives, quercetin (Q) and methyquercetins (MeQ), in cells were analyzed by HPLC-ECD. (B) Zymography (left) and immunoblot (*right*, anti-β-glucuronidase) analysis of the cultured medium of RAW264 cells. Culture medium was collected at each time point after treatment with or without LPS. (C) Immunoblot analysis of the lysates of RAW264 cells treated with or without LPS for 12 h. (D) Acidification is required for the medium β-glucuronidase activity during culturing the RAW264 cells. The β-glucuronidase activity of the cultured medium (for 8 h) at pH 5.0 or intact was determined using Q3GA as a substrate. The arrow head in the HPLC profiles (monitored at 370 nm) shows the peaks for the quercetin aglycone. (E) Acidification of the medium upon LPS treatment. The picture shows the culture plate after treatment of the RAW264 cells with (right) or without (left) treatment with LPS for 24 h. Acidification turns the medium yellow. (F) Medium lactate levels during culturing RAW264 cells in the presence of LPS, antimycin-A (AMA, 5 μg/ml), or 2-deoxyglucose (2DG, 20 mM) for 6 h determined by LC-MS/MS analysis. Blocking of glycolysis by 2DG strongly inhibited the lactate secretion. (G) Effect of lactate supplementation in the medium on the deconjugation of Q3GA. Cells were treated with Q3GA in the absence or presence of lactate (20 mM) for 4 h. Data in all bar graphs are presented as the average ± S.D. (n=3). Asterisks indicate a significant difference (*p* < 0.05). NS, not statistically significant.

Because macrophage plays an important role in mediating the inflammatory response, we investigated the effects of inflammatory response in macrophage cells on the deconjugation of Q3GA. As shown in Figure 3A, the accumulation of the deconjugated quercetin derivatives was significantly induced in the LPS-activated macrophages, showing that the inflammatory status of the macrophages is implicated in the deconjugation of the glucuronide metabolites. Zymography with a chromogenic substrate and immunoblot analysis of the cultured medium revealed that β-glucuronidase is only a major deglucuronidation enzyme secreted from the RAW264 cells (Figure 3B). It is noted that, in spite of the significant deconjugation, the LPS treatment did not induce the secretion (Figure 3B) and expression (Figure 3C) of the β-glucuronidase. The β-glucuronidase is known as a lysosomal enzyme, and therefore requires the acidic conditions for its catalytic activity. Indeed, the β-glucuronidase activity in the cultured medium was significantly enhanced by adjustment of the pH to 5 (Figure 3D). The acidification of the phenol-red containing medium, turning it yellow, was also observed during the LPS stimulation of the RAW264 cells (Figure 3E), showing that the activation of the macrophages induced the acidic conditions around the cells. It is known that lactate is a major acidic product secreted from the cells as the result of glycolysis, and its increased levels in the medium are generally used as an indicator of mitochondrial dysfunction. We then measured the lactate levels in the cultured medium by LC-MS/MS and found that the treatment with LPS, as well as a mitochondrial inhibitor antimycin-A, significantly increased lactate secretion into the medium (Figure 3F). Furthermore, the addition of lactate to the fresh medium enhanced the deconjugation of Q3GA during culturing the RAW264 cells (Figure 3G), and the inhibition of lactate production in the presence of 2-deoxyglucose resulted in the almost complete inhibition of the deconjugation (Figure S2). These results strongly suggested that the acidification around the cells through lactate secretion by mitochondrial dysfunction might assist the deconjugation of the glucuronide metabolites upon LPS activation of the macrophages.

**Figure 4:**
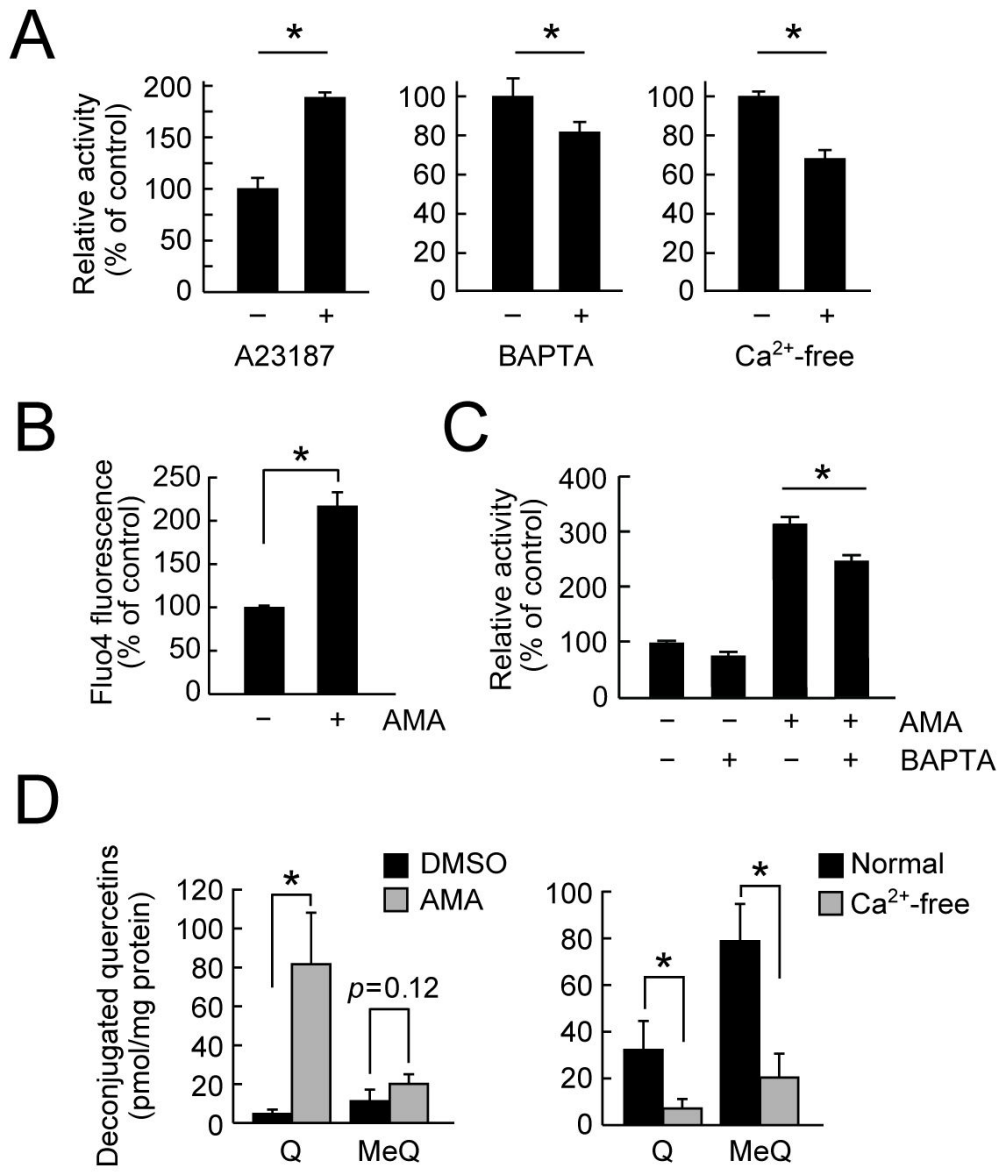
β-Glucuronidase secretion is associated with intracellular calcium ions. (A) *Left*, a calcium ionophore A23187 (10 μM, for 2 h) enhanced the β-glucuronidase activity in the medium of RAW264 cells. Reduced β-glucuronidase activity in the medium during culturing the RAW264 cells in the presence of BAPTA-AM (middle) and in Ca^2+^-free medium (right). (B) Effect of AMA treatment (for 3 h) on intracellular calcium ion levels, determined using Fluo4-AM, in the RAW264 cells. (C) Effects of AMA and BAPTA-AM on the β-glucuronidase activity in the medium during culturing the RAW264 cells. Cells were pretreated with BAPTA-AM for 1 h followed by AMA treatment for 3 h. Data in all bar graphs are presented as the average ± S.D. (n=3). Asterisks indicate a significant difference (*p* < 0.05). NS, not statistically significant. (D) Effects of AMA and Ca^2+^-free medium on the deconjugation of Q3GA. Cells were pretreated with AMA for 3 h (*left*, DMSO as control) or Ca^2+^-free DMEM medium for 8 h (*right*, normal DMEM as control) followed by incubating with Q3GA for 1 h. Quercetin derivatives accumulated in the cells were analyzed by HPLC-ECD.

It has been reported that the intracellular calcium ion is implicated in the exocytotic secretion of β-glucuronidase in macrophages [31,32]. Indeed, the treatment of macrophages with A23187, a calcium ionophore, significantly induced the β-glucuronidase activity in the medium (Figure 4A *, left*). Conversely, the β-glucuronidase activity in the medium significantly decreased upon treatment with BAPTA-AM (Figure 4A *, middle*), a cell-permeable calcium chelator, or with the calcium-free medium (Figure 4A *, right*). In these conditions, lactate secretion rates were not affected (data not shown). Consistent with the previous [33] and current observations (Figure 4B) that antimycin-A-induced mitochondrial dysfunction resulted in the increased intracellular calcium ion, the β-glucuronidase release was induced by antimycin-A in a calcium ion dependent manner (Figure 4C and Figure S3). Similarly, the deconjugation of Q3GA was significantly induced by the pretreatment of cells with antimycin-A and was inhibited by culturing in a calcium-free medium (Figure 4D). Likewise, involvement of intracellular calcium ion in the β-glucuronidase release was also observed in mouse peritoneal macrophages upon treatment with A23187, antimycin-A, or calcium-free medium (data not shown). These results suggest that mitochondrial dysfunction leads to the either enhanced acidification or β-glucuronidase release, both resulting in the increased deconjugation of glucuronide metabolites.

**Figure 5:**
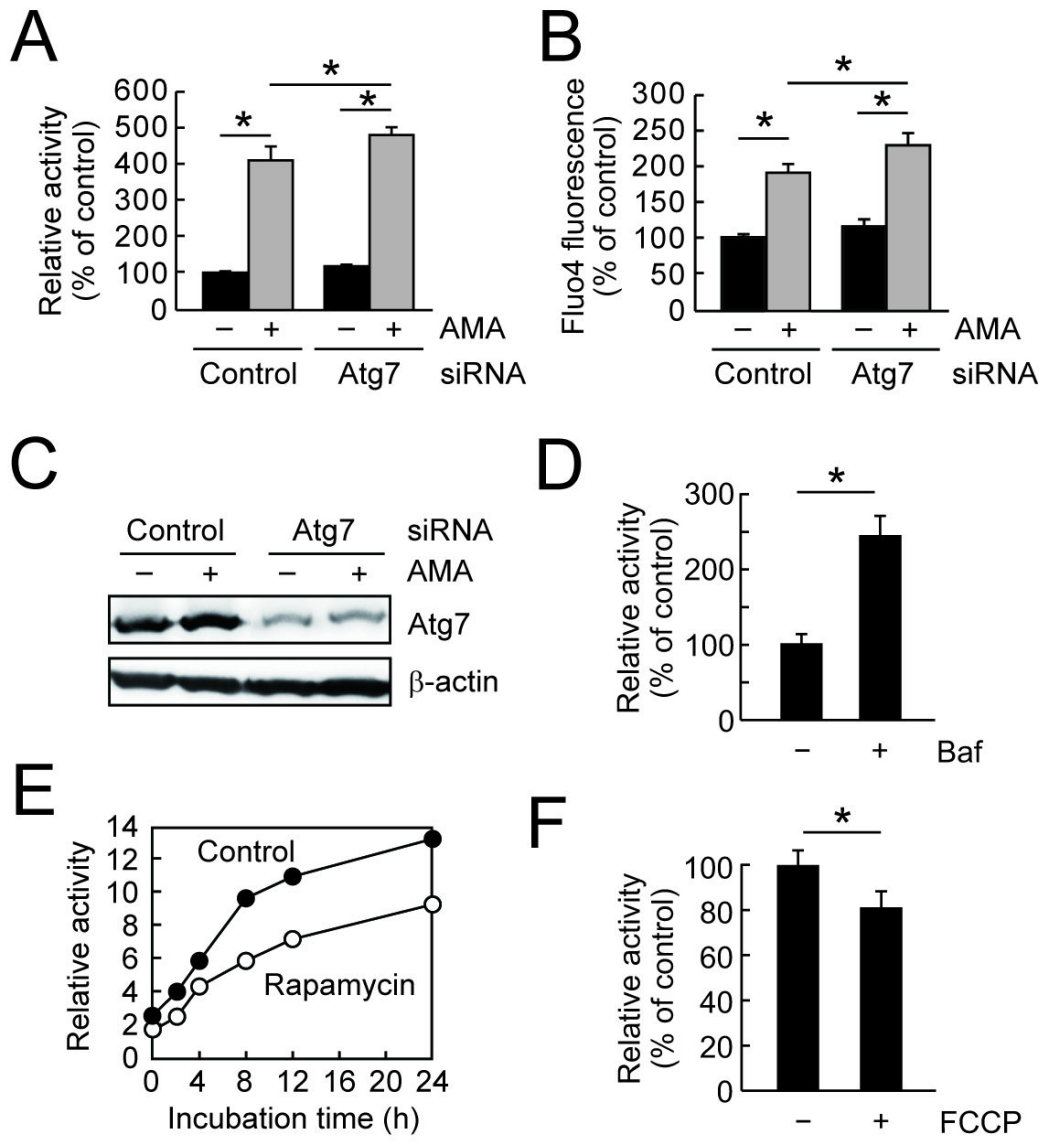
Mitochondrial quality control by autophagy is involved in the deconjugation. (A) Effects of Atg7 knockdown on the β-glucuronidase activity in the medium. (B) Effects of Atg7 knockdown on the intracellular calcium ion levels determined by Fluo4-AM probe. In *A* and *B*, RAW264 cells were transfected with siRNA for control or Atg7 and after 2 days, the cells were treated with AMA for 3 h. (C) Confirmation of siRNA knockdown of Atg7 protein by immunoblot analysis of the cell lysates used in *A* and *B*. (D-F) The β-glucuronidase activity in the cultured medium of RAW264 cells during treatment with or without chemicals: D, rapamycin (1 μM) for 0-24 h; E, carbonyl cyanide-*p*-trifluoromethoxyphenylhydrazone (FCCP, 20 μM) for 6 h; F, bafilomycin A_1_ (Baf, 200 nM) for 6 h. In D, data points represent duplicate determinations. Data in all bar graphs are presented as the average ± S.D. (n=3).

It is noted that the siRNA knockdown of Atg7, an essential gene of autophagy that maintains the mitochondrial quality, enhanced the sensitivity to the antimycin-A-induced β-glucuronidase secretion and increased intracellular calcium ion (Figures 5 A-C). Similarly, the β-glucuronidase activity was significantly induced upon treatment with bafilomycin A_1_ (Figure 5D), which prevents autophagic degradation by inhibiting the fusion of autophagosomes with lysosomes. In contrast, an autophagy inducer rapamycin significantly attenuated the β-glucuronidase activity in the medium (Figure 5E). Furthermore, carbonyl cyanide-*p*- trifluoromethoxyphenylhydrazone (FCCP), a mitochondrial uncoupler that induces mitochondria-selective autophagy (mitophagy) [34], significantly reduced the β-glucuronidase activity in the medium (Figure 5F). These results provide evidence about the important role of mitochondrial quality control by autophagy in the β-glucuronidase activity of the macrophages. We also found that the deconjugation of Q3GA was significantly attenuated in the RAW264/ρ0 cells, which are devoid of functional mitochondria but normally express β-glucuronidase (Figure S4).

### Essential role of deconjugation in the anti-inflammatory activity of a quercetin glucuronide

**Figure 6:**
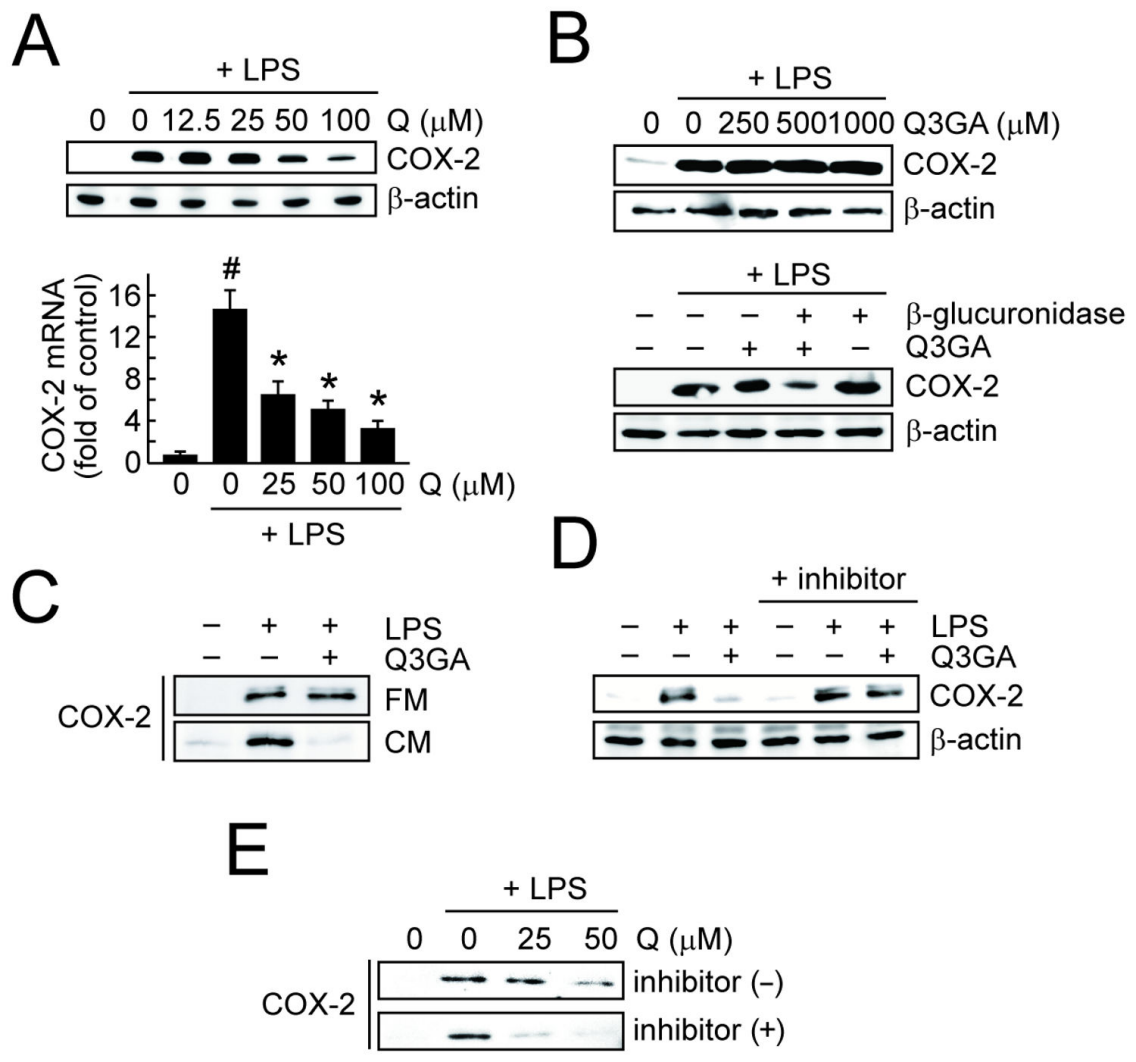
Deconjugation is essential for anti-inflammatory actions of quercetin glucuronide. (A) Effects of quercetin on the LPS-induced expression of COX-2 protein (upper) and mRNA (lower) in the RAW264 cells after 4 h. The mRNA expression of COX-2 was determined by quantitative real-time RT-PCR (GAPDH as an endogenous control). A hash symbol (#) indicates a significant difference (*p* < 0.05 vs control). Asterisks (*) indicate a significant difference (*p* < 0.05 vs the LPS treatment group). Data points represent the average ± S.D. (n=3). (B) Upper, effects of Q3GA on the LPS-induced expression of COX-2 protein (β-actin as an endogenous control) in the RAW264 cells after 4 h. Lower, effects of Q3GA (100 μM) on the LPS-induced COX-2 expression in the presence of β-glucuronidase enzyme (1 μg/ml). (C) Effects of Q3GA (100 μM) on the LPS-induced COX-2 expression in the RAW264 cells in the fresh (FM) or conditioned medium (CM). Conditioned medium was collected from 24-h cultures of the RAW264 cells. (D) Effects of Q3GA on the LPS-induced COX-2 expression in the CM in the presence of β-glucuronidase inhibitor. Experimental condition was same as *C*. (E) The inhibitory effect of quercetin on the LPS-induced COX-2 expression in the RAW264 cells was enhanced in the presence of the COMT inhibitor. Cells were incubated with LPS and quercetin in the absence or presence of the COMT inhibitor (3,5-dinitrocatechol, 10 μM) for 4 h.

The biological consequences of the deconjugation of Q3GA in the macrophages were examined for the inflammatory responses in the LPS-stimulated RAW264 cells. In this study, to examine the distinct activity between the quercetin and Q3GA, we evaluated the early phase of the COX-2 expression in the macrophages at 4 h after treatment with LPS, because no accumulation of the quercetin aglycone was observed within the 4 h-culturing of the cells in the presence of Q3GA (Figure 2B). It was clearly demonstrated that the quercetin aglycone inhibited the LPS-induced COX-2 expression of the protein (Figure 6A, *upper*) and of mRNA (Figure 6A, *lower*). In contrast, Q3GA cannot affect the COX-2 expression even at higher concentrations (Figure 6B, *upper*). However, the LPS-induced COX-2 expression was significantly inhibited in the Q3GA-treated cells in the presence of the β-glucuronidase enzyme (Figure 6B, *lower*). Similarly, the LPS-induced COX-2 expression was inhibited by the treatment with Q3GA in the conditioned medium of RAW264 cells (Figure 6C) and the inhibitory activity was abolished by the addition of a β-glucuronidase inhibitor (Figure 6D). Collectively, the secretion of β-glucuronidase into the medium from the macrophages is essential for the inhibitory effects of Q3GA on the LPS-induced COX-2 expression. It was also found that the COMT inhibitor enhanced the inhibitory effect of quercetin on the LPS-induced COX-2 expression, suggesting that quercetin itself rather than the methylquercetins is the active form for the inhibitory action (Figure 6E).

**Figure 7:**
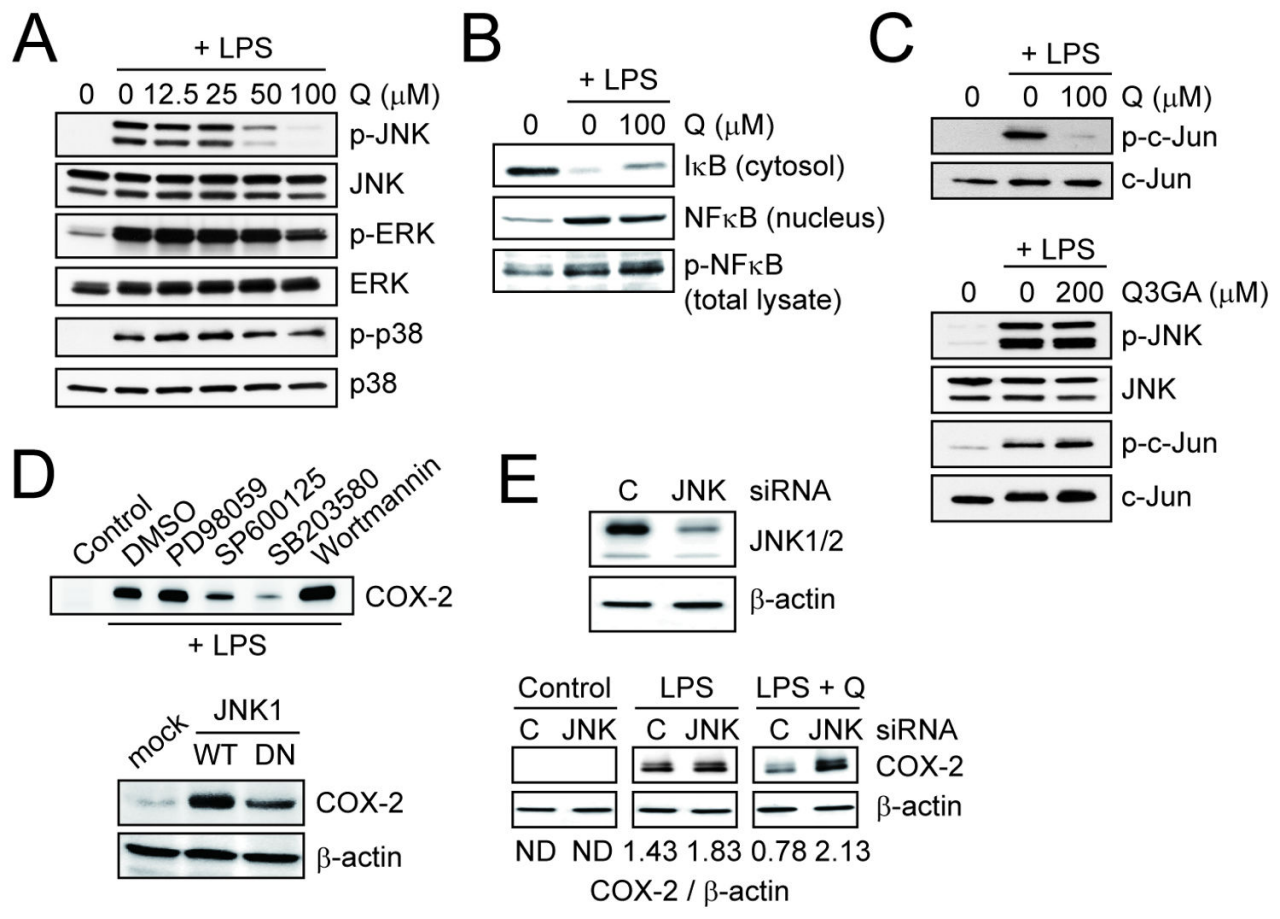
Quercetin, but not Q3GA, inhibited LPS-induced JNK activation. (A and B) Effects of quercetin on the activation of MAP kinases (A) and NFκB pathway (B) in the RAW264 cells treated with LPS for 30 min. (C) Effects of quercetin and Q3GA on the activation of JNK pathway in the RAW264 cells treated with LPS for 30 min. (D) Identification of the signaling pathways responsible for the LPS-induced COX-2 expression in the RAW264 cells. Upper, effects of inhibitors for several signaling pathways on the LPS-induced COX-2 expression in the RAW264 cells. Concentrations of inhibitors are as follows: PD98059 (for MEK/ERK), 10 μM; SP600125 (for JNK), 10 μM; SB203580 (for p38), 10 μM; and wortmannin (for PI3K, 100 nM). Cells were treated with LPS (1 μg/ml) in the presence of inhibitors for 4 h. *Lower*, overexpression of wild-type JNK1 (WT) significantly induced the COX-2 expression, whereas a dominant negative mutant of JNK1 (DN) did not. (E) Upper, immunoblot analysis of JNK1 and JNK2 (β-actin as control) to confirm the knockdown. Lower, effects of knockdown of JNK1/2 on the inhibitory actions of quercetin (100 μM) on the LPS-induced COX-2 expression in the RAW264 cells. C, control siRNA; JNK1/2, a mixture of JNK1 and JNK2 siRNA. The ratios of COX-2 to β-actin were also presented. ND, not detected.

To determine the molecular actions of quercetin regarding the inhibitory effect on the COX-2 expression, the activation of mitogen-activated protein (MAP) kinases and NF-κB was evaluated by immunoblotting. The COX-2 expression was attenuated by both pre- and post-treatments with quercetin (data not shown), excluding the possibility that quercetin artificially interacts with LPS on the cell surface and/or in the medium. Among the MAP kinase pathways, quercetin specifically attenuated the phosphorylation of JNK, but not of ERK and the p38 MAP kinases in the LPS-stimulated RAW264 cells (Figure 7A). In addition, neither the nuclear translocation nor phosphorylation of NF-κB was affected by the treatment with quercetin (Figure 7B). The phosphorylation of c-Jun, a downstream target of the phosphorylated JNK, was inhibited by quercetin but not at all by Q3GA (Figure 7C). The involvement of the JNK pathway, as well as p38 pathway, in the COX-2 expression was also demonstrated using inhibitors and overexpression experiments (Figure 7D). To further confirm the specific targeting JNK pathway, the effect of the siRNA-mediated knockdown of JNK1/2 was examined. As noted in Figure 7E, the inhibitory effect of quercetin on the LPS-induced COX-2 expression was abolished by the JNK1/2 knockdown, under which condition the COX-2 might be expressed to be significantly dependent on the p38 pathways. These results suggest that the deconjugated quercetin specifically targets the JNK pathway resulting in the inhibition of the inflammatory response in the macrophages. 

### Deconjugation of quercetin glucuronides at sites of inflammation in vivo

**Figure 8:**
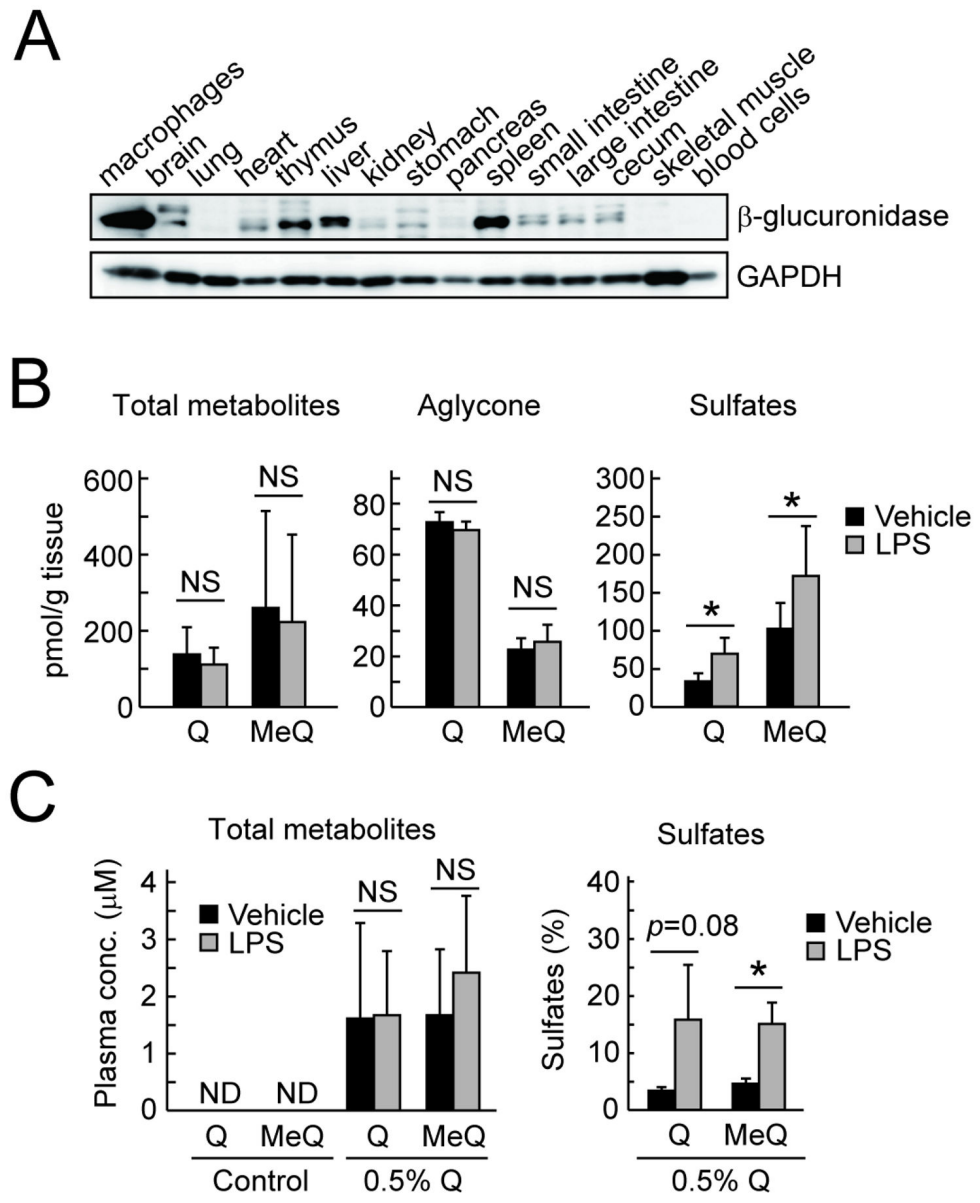
Deconjugation of quercetin glucuronides *in* *vivo*. (A) Immunoblot analysis for β-glucuronidase (GAPDH as control) in the various tissues in ICR mice. (B) Accumulation of the quercetin derivatives determined by HPLC-ECD in the spleen of ICR mice fed with 0.5% quercetin diet for 24 h followed by injection of LPS. *Left*, total metabolites; middle, aglycones; *right*, sulfates. (C) Accumulation of quercetin derivatives in the plasma of ICR mice injected with LPS. ND, not detected. Asterisks indicate a significant difference (*p* < 0.05). NS, not statistically significant. Data points represent the average ± S.D. (n=6).

Finally, we examined the *in vivo* deconjugation of quercetin glucuronides in mice. The tissue distribution of β-glucuronidase was examined by the immunoblot analysis of various tissues from ICR mice. As shown in Figure 8A, a significant expression of β-glucuronidase, as well as in the peritoneal macrophages with the most abundant expression, was observed in the thymus, liver and spleen, which may contain inflammatory cells such as lymphocytes, kupffer cells (in liver), and/or macrophages. We then examined the macrophage-mediated deconjugation of quercetin metabolites in the spleen of the 0.5% quercetin-fed mice upon LPS-induced inflammatory response. In the spleen tissue, the levels of the total quercetin metabolites and the non-conjugated quercetins were not altered regardless of the LPS treatment (Figure 8B, *left* and *middle*). It was found that the quercetin aglycone is quite unstable under biological conditions (Shiba et al., unpublished data); therefore, we speculated that it might be difficult to monitor the dynamics of the quercetin aglycone, if formed *in vivo*. However, it is noted that the sulfates of quercetin and methylquercetins were significantly increased after the injection of LPS (Figure 8B, *right*). The increase in the sulfates could reflect the deglucuronidation of the sulfoglucuronide metabolites. Similar results were also observed in the plasma, in which the quercetin sulfates were significantly increased after the LPS injection (Figure 8C). We have previously demonstrated that sulfoglucuronides are one of the major metabolites in rat plasma after intake of quercetin [35] and confirmed using LC-MS/MS the presence of sulfoglucuronides of quercetin and methylquercetins in the plasma of the quercetin-fed mice, as well as simple monoglucuronides such as Q3GA (data not shown). These results strongly revealed the *in vivo* deconjugation of the quercetin glucuronides including the sulfoglucuronides by macrophage-derived β-glucuronidase at the inflammation sites.

## Discussion

We have previously demonstrated that Q3GA specifically accumulated in macrophage cells in the foamy macrophages in the human atherosclerotic lesions, and that the macrophage cells could deconjugate Q3GA into the aglycone *in vitro* [25], whereas the molecular mechanisms and biological consequences have been unclear. In this study, an analysis of a cultured medium of RAW264 macrophage cells demonstrated that β-glucuronidase is the only major enzyme secreted from macrophages responsible for the deconjugation of phase II metabolites. We also demonstrated that LPS-stimulation of the macrophages significantly enhanced the deconjugation of Q3GA and the accumulation of the aglycones, whereas zymography and an immunoblot analysis of the medium demonstrated that the LPS treatment did not significantly increase the secretion of the β-glucuronidase enzyme (Figure 3B), suggesting that β-glucuronidase activity was enhanced upon LPS treatment. This enzyme is usually localized intracellularly in the lysosomes, which are the acidic compartments containing a variety of digestive enzymes with acidic pH optima. We found that macrophage-derived β-glucuronidase also requires acidic pH for the deconjugation of Q3GA and that the increased deconjugation of Q3GA upon LPS-stimulation could be through the enhanced acidification around the cells, which is mediated through lactate secretion associated with the mitochondrial dysfunction (Figures 3 D-G). On the other hand, although the β-glucuronidase secretion was not enhanced upon LPS treatment, the mechanism responsible for the secretion of β-glucuronidase is of importance in the regulation of deconjugation. It has been reported that intracellular calcium ion could be involved in the secretion of β-glucuronidase and other lysosomal enzymes [31,32]. Indeed, the β-glucuronidase activity was affected by intracellular calcium ion, determined using A23187, BAPTA-AM, and calcium-free medium (Figure 4). In these experiments, we utilized antimycin-A treatment as a model for the mitochondrial dysfunction in macrophages. Antimycin-A is a strong mitochondrial respiratory chain inhibitor because it completely intercepts the electrons from complexes I and II at complex III. The treatment of macrophages with antimycin-A resulted in a significant increase in the β-glucuronidase activity through both increased intracellular calcium ion and lactate secretion. It has been suggested that antimycin-A-induced reactive oxygen species might increase the intracellular calcium ions [33]. In contrast, partial inhibition of mitochondrial respiratory chain with either rotenone (complex I inhibitor) or thenoyltrifluoroacetone (TTFA, complex II inhibitor) did not induce the β-glucuronidase secretion (Figure S3), whereas they both induced lactate secretion (data not shown). Similarly, an increase in the intracellular calcium ions was scarcely observed upon LPS treatment (data not shown), although LPS treatment resulted in the increased lactate secretion (Figure 3F). These results suggest that LPS treatment partially, but not severely, inhibited the mitochondrial respiratory chain, resulting in the anaerobic lactate secretion without significant increase in the β-glucuronidase secretion. If the mitochondrial dysfunction was continuously or severely induced in the macrophages as treated with antimycin-A, the β-glucuronidase secretion, not only the lactate secretion, might also be induced through the increased intracellular calcium ions. 

Much attention has recently been paid on the mitochondrial dysfunction as a result of defects in autophagy, in particular, mitophagy. Autophagy is a cellular degradation-recycling system for aggregated proteins and damaged organelles, and its dysregulation characterized in Atg5 or Atg7-deifient mice is implicated in various age-related diseases including neurodegeneration [36,37], diabetes [38,39], and hepatocarcinoma [40,41]. Recent studies further demonstrated that mitochondrial dysfunction derived from autophagy/mitophagy impairment in the inflammatory cells could be implicated in the induction of chronic inflammation [42-44]. We have demonstrated that (i) the autophagy impairment by siRNA knockdown of Atg7 induced the β-glucuronidase activity in macrophages (Figure 5A), and that (ii) two different types of autophagy inducers, rapamycin and FCCP, reduced the β-glucuronidase activity (Figures 5 D and E). It has been reported that Atg7 knockout cells exhibited a defect in mitochondrial respiration and a compensatory increase in basal glycolytic rates [39]. These observations suggested that autophagy impairment could be a trigger for mitochondrial dysfunction that induces the secretion and activity of β-glucuronidase. It has also been reported that quercetin could accumulate and act in the mitochondria of Jurkat cells [45]. A recent paper has demonstrated that polyphenolic compounds could also induce autophagy [46]. Furthermore, we found the involvements of protein kinase C and MAP kinases, which are also the inhibitory targets of quercetin and other flavonoids [47], in the secretion of β-glucuronidase from macrophages (Miki et al., unpublished data). Based on these observations, our results indicated a pathway as if inflammatory macrophages recruit the glucuronide metabolites of flavonoids to inhibit their inflammation. Generally, inflammation is necessary for elimination of a pathogen and damaged cells, but needs to be tightly controlled. Thus, the interaction between flavonoid glucuronides and macrophages might contribute to the attenuation of excessive inflammation in macrophages.

**Figure 9:**
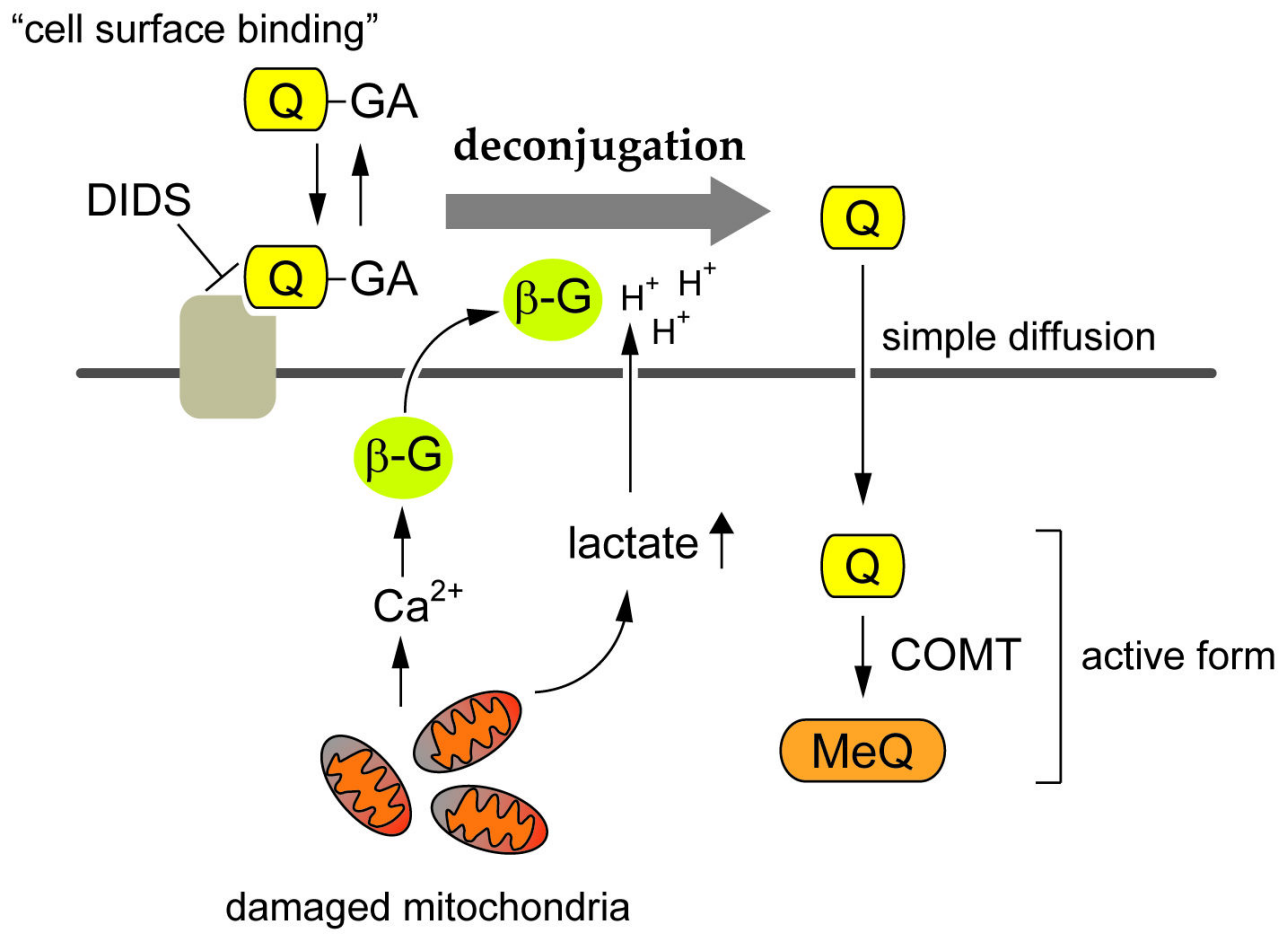
Scheme for the proposed mechanism of the deconjugation of quercetin glucuronides by macrophages. Quercetin glucuronides (Q-GA) bound to the DIDS-sensitive cell surface proteins of macrophages were readily doconjugated into the aglycone (Q) by the β-glucuronidase (β-G) activity under acidic conditions derived from increased lactate secretion associated with mitochondria dysfunction. The β-glucuronidase secretion was promoted by increased intracellular calcium ions (Ca^2+^). The deconjugated aglycones (Q and the methylated form, MeQ) accumulated in the cells could be the active form for the anti-inflammatory and anti-atherosclerotic activities.

We demonstrated that Q3GA accumulates on the cell surface of the macrophages through the anion binding of the phenolic group(s) of Q3GA to the DIDS-sensitive cell surface proteins (Figure 1). However, the cell surface binding of Q3GA itself failed to inhibit the LPS-induced intracellular signaling pathways in the absence of the deconjugation activity. In contrast, quercetin aglycone, much more hydrophobic than Q3GA, could accumulate inside the cells and significantly inhibited the LPS-stimulated intracellular signaling, in particular the JNK pathway. Thus, the anion binding of Q3GA to the cell surface proteins of macrophages might be important because Q3GA could efficiently be deconjugated into the aglycone on the cell surface, where the relative concentrations of both β-glucuronidase and lactate are presumed to be higher. Therefore, the Q3GA-binding cell surface proteins might play a role as a scaffold of Q3GA for the following deconjugation. The specific Q3GA-binding proteins on the cell surface could not be identified in this study. To identify them, the proteomic approaches combined with the specific antibodies to Q3GA and to the DIDS are now in progress in our laboratory. The molecular actions of quercetin glucuronides proposed in this study are illustrated in Figure 9.

In spite of the potent mutagenicity and cytotoxicity of quercetin *in vitro*, they have not been confirmed *in vivo* following the oral administration of quercetin [48]. The safety of orally administered quercetin *in vivo* may reflect the fact that quercetin is almost completely metabolized during absorption [19]. It is generally accepted that phase II detoxification converts hydrophobic chemicals into hydrophilic metabolites, and then helps to limit the entry of the chemicals into cells. Indeed, the entry of Q3GA into the macrophage cells was limited only to the cell surface. Many papers have previously suggested the potential roles of the conjugated metabolites of dietary flavonoids on the prevention for inflammatory- and age-related diseases. However, in most *in vitro* studies [18], the biological activity of the flavonoid conjugates was examined after longer incubation periods (e.g., for 24 h), which might lead to the deconjugation of the metabolites in some cell types, such as macrophages. We also demonstrated similar results in which the treatment of the RAW264 cells [25] or differentiated THP-1 cells (Nishikawa et al. unpublished data) with Q3GA for 24 h significantly attenuated the gene expression of a class-A scavenger receptor in a β-glucuronidase-dependent manner. Furthermore, we found that the quercetin aglycone was quite unstable under the biological conditions and the inhibitory activity for the COX-2 expression was almost completely attenuated after the degradation of quercetin (Shiba et al., unpublished data). In contrast, quercetin conjugates including Q3GA are quite stable under the biological conditions [49]. These observations suggest that the macrophage-derived deconjugation plays an important role in transiently providing bioactive/unstable aglycones in the biological fluids, in particular, within the sites of inflammation. The selective deconjugation within the sites of inflammation might ensure the safety of flavonoids in normal tissues. 

We finally demonstrated that the injection of LPS into mice resulted in the increased accumulation of quercetin sulfates in the spleen and plasma *in vivo* (Figure 8). This result may reflect the deglucuronidation of sulfoglucuronides at the inflammatory sites. It has been reported that the sulfated metabolites of quercetin, such as quercetin-3’-sulfate and quercetin-sulfoglucuronides, are also the major metabolites in the plasma and urine of human [19,50]. In contrast to the significant β-glucuronidase activity, the sulfatase activity was scarcely observed in macrophage cells (Figure 2). Thus, the quercetin sulfates, as well as quercetin, could be the major products of macrophage-mediated deconjugation at inflammatory sites. Although the biological activity of quercetin sulfates has not yet been examined in this study, they could affect the intracellular events by accumulating inside the cells, because the sulfates are generally more hydrophobic than the glucuronides. 

In summary, we have now demonstrated the molecular basis of the secretion and activity of β-glucuronidase, which is the key molecule for the biological activity of the phase II metabolites of flavonoids *in vivo*. The recruitment and the following deconjugation of Q3GA on the cell surface of the macrophages, which could be set up through the mitochondrial dysfunction, were essential for the anti-inflammatory actions of Q3GA. These observations showed that the macrophage-mediated deconjugation provides a selective anti-inflammatory activity of quercetin glucuronides.

## Supporting Information

Figure S1 **Detection of DIDS-modified proteins using a newly developed monoclonal antibody.** (A) Cross-reactivity of anti-DIDS monoclonal antibody (mAb16D4) to the DIDS- or SITS-treated bovine serum albumin (BSA), determined by ELISA. (B) Immunoblot analysis of the DIDS-treated BSA with mAb16D4. (C) Immunoblot analysis of the cell lysates of DIDS-treated RAW264 cells with mAb16D4. Cells were treated with DIDS (1 mM) for 15 min.
(PDF)

Figure S2 **Inhibition of the deconjugation of Q3GA in the presence of 2-deoxyglucose during culturing of RAW264 cells.** Cells were treated with Q3GA (50 μM) in the absence (B) or presence (C) of 2-deoxyglucose (2DG, 20 mM) for 8 h. The quercetin derivatives were extracted in the cell lysates and analyzed by HPLC with the detection at 370 nm. (A) Authentic standards, quercetin and a methylquercetin (isorhamnetin).
(PDF)

Figure S3 **β-Glucuronidase release from RAW264 cells upon treatment with mitochondrial inhibitors.** (A) Immunoblot analysis of the cultured medium of RAW264 cells treated with each mitochondrial inhibitor (rotenone 10 μM, TTFA 0.5 mM, or antimycin-A (AMA) 50 μg/ml) for 3 h. (B) The β-glucuronidase activity in the medium (same samples in A). Data in all bar graphs are presented as the average ± S.D. (n=3). Asterisks indicate a significant difference (*p* < 0.05).
(PDF)

Figure S4 **The deconjugation of Q3GA in the RAW264 cells lacking mitochondrial DNA (ρ0 cells).** (A) Cells were treated with Q3GA (50 μM) for 8 h and the quercetin derivatives in the cells were analyzed by HPLC-ECD. (B) Immunoblot analysis for β-glucuronidase in the lysates of RAW264 and the ρ0 cells. Asterisks indicate a significant difference (*p* < 0.05). NS, not statistically significant. Data in all bar graphs are presented as the average ± S.D. (n=3).
(PDF)
