# Supplementary figures and images for: Mitochondrial Dysfunction Leads to Deconjugation of Quercetin Glucuronides in Inflammatory Macrophages
Source: PLoS One. 2013 Nov 19;8(11):e80843. doi: 10.1371/journal.pone.0080843 (PMC3834324; doi:10.1371/journal.pone.0080843)

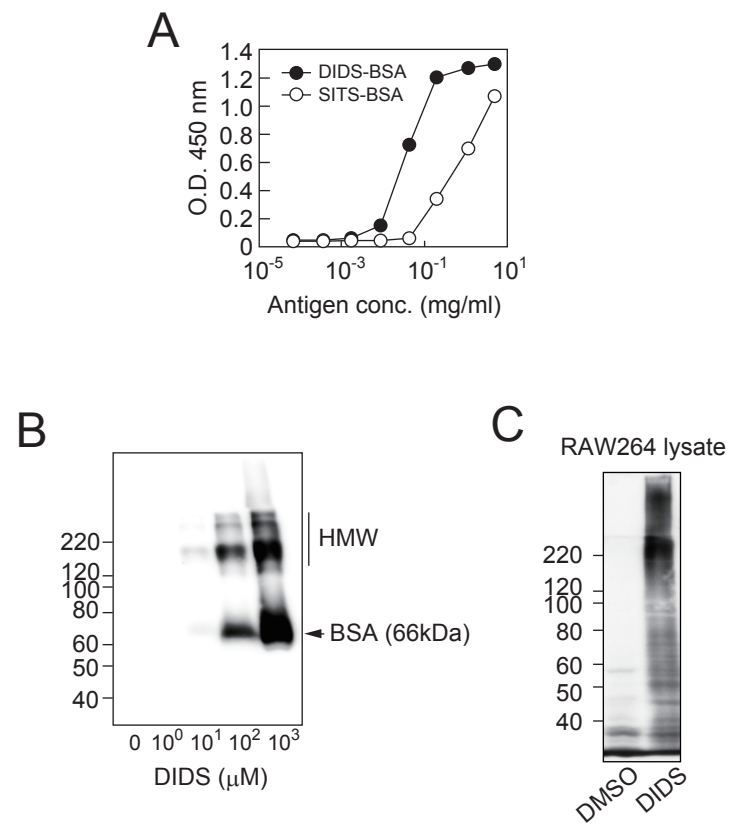

Fig. S1  
Ishisaka et al

Supplement: Figure S1 — Detection of DIDS-modified proteins using a newly developed monoclonal antibody. (A) Cross-reactivity of anti-DIDS monoclonal antibody (mAb16D4) to the DIDS- or SITS-treated bovine serum albumin (BSA), determined by ELISA. (B) Immunoblot analysis of the DIDS-treated BSA with mAb16D4. (C) Immunoblot analysis of the cell lysates of DIDS-treated RAW264 cells with mAb16D4. Cells were treated with DIDS (1 mM) for 15 min. (PDF) [file pone.0080843.s001.pdf]

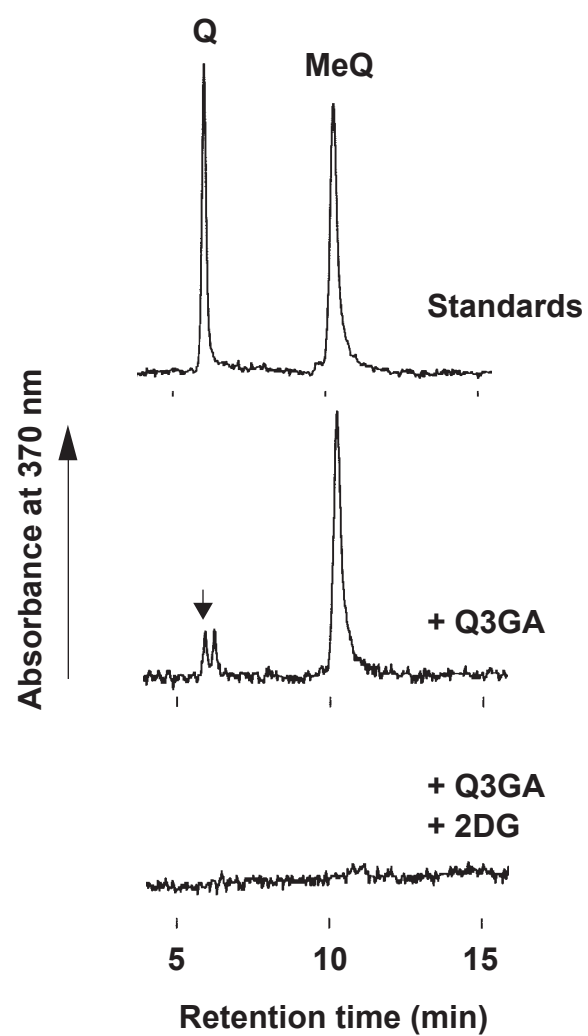

Fig. S2  
Ishisaka et al

Supplement: Figure S2 — Inhibition of the deconjugation of Q3GA in the presence of 2-deoxyglucose during culturing of RAW264 cells. Cells were treated with Q3GA (50 μM) in the absence (B) or presence (C) of 2-deoxyglucose (2DG, 20 mM) for 8 h. The quercetin derivatives were extracted in the cell lysates and analyzed by HPLC with the detection at 370 nm. (A) Authentic standards, quercetin and a methylquercetin (isorhamnetin). (PDF) [file pone.0080843.s002.pdf]

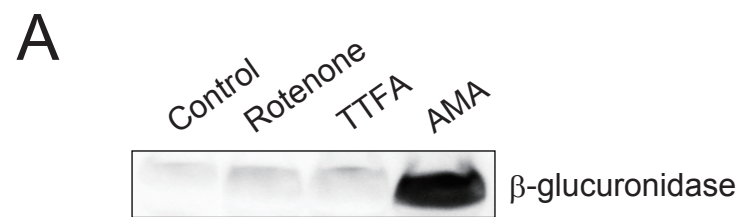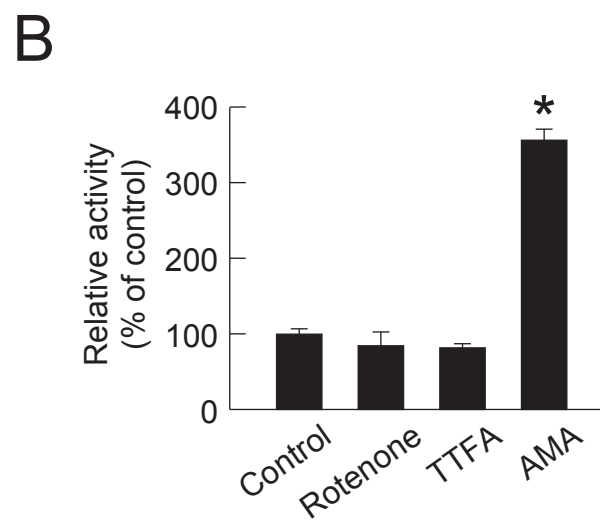

Fig. S3  
Ishisaka et al

Supplement: Figure S3 — β-Glucuronidase release from RAW264 cells upon treatment with mitochondrial inhibitors. (A) Immunoblot analysis of the cultured medium of RAW264 cells treated with each mitochondrial inhibitor (rotenone 10 μM, TTFA 0.5 mM, or antimycin-A (AMA) 50 μg/ml) for 3 h. (B) The β-glucuronidase activity in the medium (same samples in A). Data in all bar graphs are presented as the average ± S.D. (n=3). Asterisks indicate a significant difference (p < 0.05). (PDF) [file pone.0080843.s003.pdf]

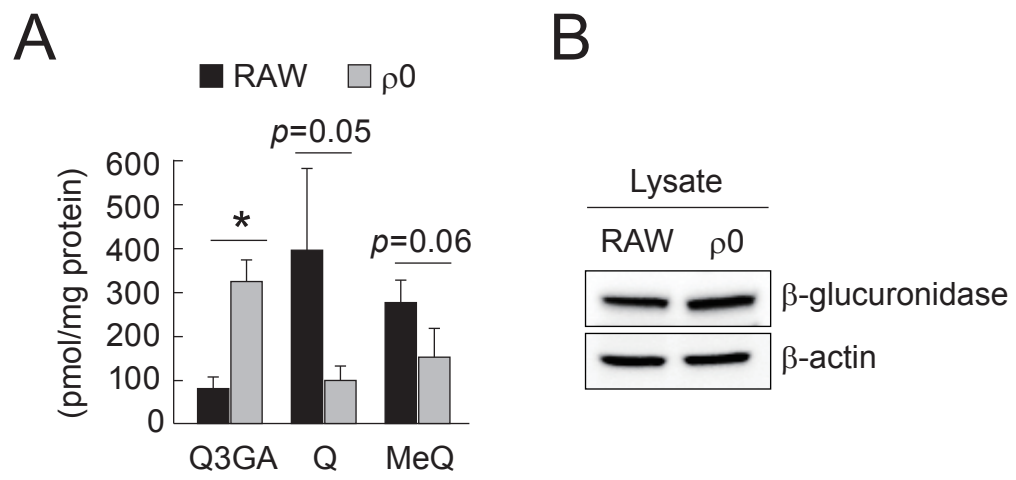

Fig. S4  
Ishisaka et al

Supplement: Figure S4 — The deconjugation of Q3GA in the RAW264 cells lacking mitochondrial DNA (ρ0 cells). (A) Cells were treated with Q3GA (50 μM) for 8 h and the quercetin derivatives in the cells were analyzed by HPLC-ECD. (B) Immunoblot analysis for β-glucuronidase in the lysates of RAW264 and the ρ0 cells. Asterisks indicate a significant difference (p < 0.05). NS, not statistically significant. Data in all bar graphs are presented as the average ± S.D. (n=3). (PDF) [file pone.0080843.s004.pdf]
